# Autopsy of a failed trial part 1: A qualitative investigation of clinician's views on and experiences of the implementation of the DAISIES trial in UK‐based intensive eating disorder services

**DOI:** 10.1002/erv.2975

**Published:** 2023-03-23

**Authors:** Matthew Phillips, Başak İnce, Hannah Webb, Bethan Dalton, Catherine McCombie, Madeleine Irish, Daniela Mercado, Gemma Peachey, Zohra Zenasni, Hubertus Himmerich, Paul Robinson, Jon Arcelus, Sarah Byford, Janet Treasure, Sabine Landau, Vanessa Lawrence, Ulrike Schmidt

**Affiliations:** ^1^ Section of Eating Disorders Department of Psychological Medicine Institute of Psychiatry, Psychology, and Neuroscience King's College London London UK; ^2^ Department of Health Service and Population Research Institute of Psychiatry, Psychology & Neuroscience King's College London London UK; ^3^ South London and Maudsley NHS Foundation Trust Maudsley Hospital London UK; ^4^ Department of Biostatistics and Health Informatics Institute of Psychiatry, Psychology & Neuroscience King's College London London UK; ^5^ Division of Medicine University College London London UK; ^6^ Institute of Mental Health University of Nottingham Jubilee Campus Nottingham UK

**Keywords:** anorexia nervosa, barriers to recruitment, implementation research, intensive treatment, qualitative research

## Abstract

**Objective:**

The DAISIES trial, comparing inpatient and stepped‐care day patient treatment for adults with severe anorexia nervosa was prematurely terminated in March 2022 due to poor recruitment. This qualitative study seeks to understand the difficulties faced during the trial by investigating stakeholders' views on and experiences of its implementation.

**Method:**

Semi‐structured interview and focus group transcripts, and trial management and oversight group meeting minutes from May 2020‐June 2022 were analysed using thematic analysis. Participants were 47 clinicians and co‐investigators involved with the DAISIES trial. The Non‐Adoption, Abandonment, Scale‐up, Spread, and Sustainability (NASSS) framework was applied to the interpretive themes to classify barriers and facilitators to implementation.

**Results:**

Five themes were identified: incompatible participation interests; changing standard practice; concerns around clinical management; systemic capacity and capability issues; and Covid‐19 disrupting implementation. Applying the NASSS framework indicated the greatest implementation challenges to arise with the adopters (e.g. patients, clinicians), the organisational systems (e.g. service capacity), and the wider socio‐political context (e.g. Covid‐19 closing services).

**Conclusions:**

Our findings emphasise the top‐down impact of systemic‐level research implementation challenges. The impact of the Covid‐19 pandemic accentuated pre‐existing organisational barriers to trial implementation within intensive eating disorder services, further limiting the capacity for research.

## Highlights

- The paper highlights the particular challenges to research implementation that arise within an NHS intensive service context.
- Applying an implementation science framework to the interpretive themes indicated that challenges to implementation spanned the individual to the systemic level, with the latter posing greater barriers and impacting implementation success in other areas.
- The results suggest that future research into intensive treatment needs to better accommodate patient preferences and emphasise clinician‐researcher relationships and the alignment of clinical and research teams.

## 1 INTRODUCTION

Estimates suggest that up to 25% of all clinical trials are prematurely terminated, mostly due to poor recruitment (Davidović et al., 2020; Kasenda et al., 2014; Williams et al., 2015; Wortzel et al., 2020). Early termination of a trial represents an undesirable return on research resource investment and has ethical implications for participants who believed they would be contributing socially useful data (Malmqvist et al., 2011); despite this, the majority of terminated trials are unpublished (Kasenda et al., 2014; Williams et al., 2015), preventing lessons from being learnt. Disseminating the results of and reasons behind terminated trials is important in informing future research.

The DAISIES trial was a UK‐based randomised controlled multi‐centre open‐label parallel group non‐inferiority trial of the clinical effectiveness, acceptability, and cost‐effectiveness of a stepped‐care day patient (DP) approach versus inpatient (IP) treatment‐as‐usual for anorexia nervosa (AN) in adult specialist eating disorder (ED) services. In both IP and stepped‐care DP treatment, patients receive multidisciplinary treatment at a specialist ED unit until they reach a healthy weight and normalise their eating, or get as close to this point as possible. In IP, patients stay at the unit until discharge, whereas in DP they attend 4–5 days a week, returning home for weeknights and weekends, with the option of a brief IP admission for medical stabilisation. Participants were recruited from intensive ED services in the UK. Full details on study rationale, design, and procedures can be found in the protocol (Irish et al., 2022). In brief, AN is a serious mental health condition associated with adverse psychological and physical consequences, pronounced functional impairment, and high mortality (Treasure et al., 2020; Zipfel et al., 2015). The acuity of presentations appears to be rising in the UK, indicated by increasing hospital admissions (NHS Digital, 2020); however, little is known about the comparative effectiveness of intensive treatment approaches for those most ill (Beat, 2019). Generally, funding for ED research is low, and clinical trials are sparse (Woelbert et al., 2021; Wortzel et al., 2020). This research gap and treatment need motivated the funding of the DAISIES trial.

Trial set‐up began in January 2020, and recruitment opened in November 2020. The timeline of the trial coincided with the onset of the Covid‐19 pandemic in the UK, where ED admissions increased whilst intensive services reduced in capacity or closed (Ayton et al., 2022; NHS Digital, 2020), including those which were planned recruiting sites for the trial (İnce et al., 2023). In total, 53 patients were approached, of which 15 enroled, where the recruitment target was 386. The trial was prematurely terminated in March 2022 due to poor recruitment.

Given the pronounced need for intensive treatment and relative scarcity of trials on intensive treatment approaches for AN, it is imperative that the difficulties faced during the DAISIES trial are fully explored, rather than simply attributed to the impact of Covid‐19. The present study therefore seeks to investigate in‐depth the views and experiences of stakeholders on implementing the DAISIES trial, through using a qualitative methodology and applying a theoretical implementation framework to the results. For an overview of the outcomes of the trial and a researcher‐led description of difficulties, see İnce et al. (2023); for qualitative investigations of clinicians’ experiences of delivering intensive ED services before and during the Covid‐19 pandemic, see Webb et al. (2022a, 2022b).

The present study applied the Non‐Adoption, Abandonment, Scale‐up, Spread, and Sustainability (NASSS) framework (Greenhalgh & Abimbola, 2019), an evidence‐based framework that captures and evaluates the success of the implementation of healthcare technology projects in terms of their complexity (in this context, the DAISIES trial was conceptualised as the ‘technology’). The NASSS framework has previously been applied in qualitative analyses to evaluate implementation of healthcare technologies or interventions (Banck & Bernhardsson, 2020; Cartledge et al., 2022), though its application to a clinical trial is novel. The NASSS framework consists of seven domains in implementation projects where complexity can lie: the condition, the technology itself, the value proposition, the adopters (e.g. patients and clinicians), the organisation, the wider socio‐political context, and the evolution of each domain over time. Complexity in multiple domains limits the likelihood of successful implementation (Greenhalgh et al., 2017); exploring these domains can therefore aid the identification of factors impacting implementation. The research question was:

‐ What were stakeholders' views on and experiences of implementing the DAISIES trial?

## 2 METHODS

Ethical approval was granted by the Wales Research Ethics Committee 5 (Reference: 20/WA/0072). Data collection for this study was conducted as part of the process evaluation for the DAISIES trial (Irish et al., 2022). A qualitative design was used, underpinned by a critical realist philosophical orientation (Bhaskar, 2016; Fletcher, 2017) which separates ontology and epistemology, positing an objective reality understandable via subjective socially constructed knowledge. This study therefore combines subjective accounts of experience with theory (the NASSS framework) in the analysis to better understand the mechanisms underlying the implementation of the DAISIES trial.

### 2.1 Participants

Participants were 47 purposively‐sampled professionals who were involved in the DAISIES trial. Twenty‐six were clinicians working in intensive ED services at planned DAISIES sites (see Table 1), many of whom were DAISIES site leads (i.e. senior clinicians responsible for recruitment and oversight of the DAISIES trial at their sites) or recruitment champions (i.e. clinical staff with recruitment and data collection responsibilities). Many of these clinicians were therefore known to the researchers conducting the interviews. Clinicians worked across a range of specialist ED settings and roles, such as consultant psychiatrists, nurses, and psychologists. The majority had 10+ years of ED experience. Twenty‐one were DAISIES co‐investigators, members of the DAISIES Trial Management Group (TMG) and Trial Steering Committee (TSC), a team of experienced trialists, junior researchers, and lived experience experts. TMG meetings were held every 2 months for the duration of the trial, and concerned oversight of the day‐to‐day running of the trial. TSC meetings were held once yearly and concerned the trial's overall supervision (National Institute for Health and Care Research, 2022). A frequency table of member roles can be found in Table 2.

**TABLE 1:** Participant ID, work setting, role, and years of eating disorder experience of interviewed clinicians.

Participant ID	Work setting	Role	Years of experience in EDs
P1	OP	Consultant psychiatrist	10+
P2	IP/DP	Consultant psychiatrist	5–10
P3^a^	IP	Consultant psychiatrist	10+
P4^a^	DP	Occupational therapist	5–10
P5	OP/DP	Consultant psychiatrist	10+
P6	OP	Clinical service manager	10+
P7	DP	Nurse specialist	0–5
P8^a^	IP	Consultant psychiatrist	0–5
P9	IP/DP	Nurse therapist	10+
P10	OP/DP	Dietician	10+
P11	OP/DP	Nurse specialist	0–5
P12	IP	Counselling psychologist	0–5
P13	DP	Mental health nurse	0–5
P14	DP	Assistant psychologist	0–5
P15	IP	Counselling psychologist	10+
P16	DP	Assistant psychologist	0–5
P17	DP	Mental health nurse	0–5
P18	OP/DP	Occupational therapist	0–5
P19	DP	Occupational therapist	0–5
P20	OP/DP	Consultant psychiatrist	10+
P21	IP	*Missing*	*Missing*
P22	IP/DP	Consultant psychiatrist	10+
P23	OP	Clinical lead	10+
P24	IP/DP	Consultant psychiatrist	10+
P25	IP/DP	Consultant psychiatrist	10+
P26	IP/DP	Consultant psychiatrist	10+

Abbreviations: DP, Day Patient; ED, Eating Disorder; IP, Inpatient; OP, Outpatient.

^a^ Participated in both interview periods.

**TABLE 2:** Frequencies of Trial Management Group and Trial Steering Committee member roles.

Role	Frequency
Junior researcher	8
Senior researcher	4
Lived experience expert	3
Statistician	3
Health economist	2
Qualitative social scientist	1

### 2.2 Data collection

The data corpus consisted of data from different sources over the course of the DAISIES trial; namely, two sets of transcripts of semi‐structured interviews and focus groups with clinicians conducted between May 2020 to June 2021 and April to June 2022, and the written meeting minutes of all TMG and TSC meetings held during the trial. The first interview period therefore covers study set‐up and early recruitment phases, and the second period covers part of the closedown phase. In total, the data corpus consisted of 22 interview transcripts, two focus group transcripts, and 19 meeting minutes (Appendix 1).

The semi‐structured interviews held between May 2020 and June 2021 (for the respective topic guide, see Appendix 2) were conducted by researchers BD, DM, MI, and GP. For the present study, only data relating to the implementation of the DAISIES trial were analysed (for further detail and analysis of the broader data, see Webb et al., 2022a, 2022b). The semi‐structured interviews and focus groups held between April and June 2022 were conducted by researchers MP and Bİ. The topic guide for these (Appendix 3) was designed by researchers MP, Bİ, CM, VL, US, and concerned participants' thoughts and feelings surrounding the closure of the DAISIES trial, experiences of its implementation within their services, and the perceived learning from the trial. This topic guide was broadly organised according to the NASSS framework and informed by answers given in previous semi‐structured interviews with clinicians and the results of prior patient and public involvement work with patients in IP and DP services (İnce et al., 2023). Clinicians were encouraged to express both positive and negative views and assured of their anonymity. Several clinicians participated in interviews during both interview periods; many of the initial interviewees were re‐approached after trial closure, to capture possible changes in opinion. TMG and TSC meeting minutes summarised key meeting activities and discussions, and were transcribed by a junior researcher.

### 2.3 Analysis

Data were analysed in NVivo 12 in accordance with the framework of thematic analysis (Table 3; Braun & Clark, 2006, 2021). After data familiarisation, researchers MP and Bİ constructed an initial coding framework organised according to NASSS domains, where each domain was a category with associated codes. Consistent with a critical realist stance, coding was deductive and inductive; the NASSS framework was used as a guide to focus the analysis towards identifying the complexity of implementing DAISIES, but development of new codes for the data was ongoing. This coding framework was applied by MP to the dataset for analysis and continually refined throughout the analytic process through meetings between MP, Bİ, and VL, to discuss developing understandings of the dataset. Bİ independently coded 7 interviews and 3 meeting minutes, enhancing the trustworthiness of the findings through facilitating ongoing conversations and critique of how the framework should be applied. MP and Bİ kept reflexive journals throughout to draw upon and reflect on how their knowledge of ED treatment and their experiences of working on the DAISIES trial and with people with EDs in outpatient and intensive treatment settings may have influenced their approaches to the data and analysis.

**TABLE 3:** Framework of thematic analysis.

Phase	Description
1. Data familiarisation	Transcription; reading and re‐reading data; noting initial ideas
2. Generating initial codes	Coding interesting data systematically; collating data relevant to each code
3. Searching for themes	Collating codes into potential themes; gathering all relevant data
4. Reviewing themes	Checking congruence between themes and coded extracts and data set; generating a thematic map
5. Defining and naming themes	Thematic refinement and refinement of overall analytic narrative
6. Producing the report	Selection and analysis of extracts; writing of results and interpretation

As analysis developed and more codes were derived from the data, the utility of using the NASSS framework to guide analysis diminished. Codes spanned multiple NASSS domains, and conceptually related items often had to be separated artificially across different domains. The decision was therefore made to abandon the NASSS framework during phases 3, 4, and 5, allowing for theme generation and refinement to accord with researcher‐identified patterns of meaning. The NASSS framework was then applied retrospectively to the final themes and sub‐themes. Researchers MP and Bİ mapped themes into the NASSS domains, classifying them as either barriers or facilitators of trial implementation (e.g. barriers to recruitment, facilitators of integrating research processes in clinical practice). The complexity of each domain was then determined: either simple (straightforward, predictable, few components), complicated (multiple interacting components or issues), or complex (dynamic, unpredictable, not easily disaggregated into constituent components; Greenhalgh et al., 2017).

## 3 RESULTS

The analysis sought to understand stakeholders' views on and experiences of implementing the DAISIES trial. Five overarching themes were generated from the data: Incompatible participation interests; Changing standard practice; Concerns around clinical management; Systemic capacity and capability issues; Covid‐19 disrupting implementation.

### 3.1 Theme 1: Incompatible participation interests

#### 3.1.1 The perceived appeal of DAISIES to participants

Many clinicians interviewed during the early stages of the trial expressed optimism that participating in DAISIES would appeal to patients and carers. Clinicians commonly felt that some of the motivation to participate would stem from altruism, to *‘give something back’* (P6), and that participants would find it ‘*quite exciting’* (P13) to contribute to a trial with the potential to improve intensive care for AN.

It might give them a sense of purpose as well. To do something good for the world and people with anorexia and, yes, for other patients and for the future. (P5)

However, several clinicians reflected on aspects of DAISIES that might be perceived as less appealing to participants, including completing follow‐up assessments, which were seen as potentially *‘cumbersome’* (P11) and *‘a chore’* (P2), and intensive treatment being decided by randomisation, as *‘folk are more reluctant to accept a random choice’* (P26).

#### 3.1.2 Difficulty pitching the trial to patients

Many clinicians recognised the difficulty of pitching the trial to patients. Towards the start of the trial, some clinicians expressed apprehension that the trial might ‘*come across as something that is restricting them* [patients]’ (P5) and that randomisation may therefore be perceived negatively. Once participant recruitment did begin, clinicians commonly reported difficultly engaging with patients, who were often recruited from IP services where presentations were most acute. Several clinicians raised concerns over the mental capacity of patients, *‘wondering how much they were able to weigh up the pros and cons’* (P21) of participation. Patient ambivalence and anxiety towards treatment were also mentioned by some as barriers to recruitment, with one clinician noting ambivalence to be a *‘key challenge’* (P26).

It's a big thing to ask people at a point where they're unwell and anxious about should I take part now or not. (P1)

These recruitment difficulties highlighted the importance of clear communication with participants about the trial and its impact on their treatment, with several clinicians noting a need to ensure *‘that our patients’ expectations are clear and we’re not trying to meet expectations that we’re not onboard with’* (P14).

#### 3.1.3 Strong preference for day treatment

Most clinicians felt that patients would have a pronounced preference for DP over IP treatment. The stepped‐care DP treatment arm was therefore perceived as desirable to patients, for whom the shorter IP admission prior to step‐down was suggested to be *‘a positive’* (P9), with one clinician stating that this *‘would be their* [the patients’] *ideal’* (P12). There was a widespread perception that for some patients, the trial would be seen *‘as an opportunity to avoid inpatient admission’* (P8). While some clinicians saw this as motivating and a recruitment opportunity, others expressed concern about consequent patient engagement in day treatment.

The ones who didn't want to be on the ward embraced the study and it gave them hope (P21)

A strong preference for day services was also seen by many clinicians as a deterrent for participation, with patients ‘*not willing to take the risk on getting stuck in the inpatient arm’* (P22). TMG meeting minutes towards the end of the trial reported strong treatment preference as *‘a very common reason’* (TMG15) for patients declining to participate.

### 3.2 Theme 2: Changing standard practice

#### 3.2.1 The appeal of changing standard practice

Clinicians commonly identified DAISIES as an important trial, addressing necessary questions about intensive treatments for AN that could contribute to evidence‐based practice and provide ‘*some data with which to guide our decision making…* [as there is] *almost no data about day patient’* (P26). Many clinicians considered he knowledge gap around intensive treatments to be acute:

We are definitely in desperate need of some sort of standardisation and better understanding of what hospitals are for. What are the treatments that we need to provide? (P24)

Clinicians therefore commonly viewed the DAISIES trial as *‘very exciting’* (P12), but also ‘*anxiety‐provoking’* (P16) due to the uncertainty of changing standard practice. Despite this anxiety, several clinicians noted that being part of the DAISIES trial had facilitated discussions amongst their team surrounding their clinical work.

Even before the results are available we’ll think about the treatment in a different way (P3)

In particular, the introduction of a comprehensive traffic light risk assessment tool to support clinical decision making in the stepped‐care DP arm (for details, see Irish et al., 2022) was felt by many clinicians to be ‘*helpful for the team’* (P19) and *‘an excellent and quite objective way to assess the risk of people’* (P3).

#### 3.2.2 Changes in workloads and roles

Many clinicians wondered about how their team's work might change when implementing the trial. Some DP clinicians mentioned that ‘*depending on the severity of, like, particular patients, it can increase workload’* (P17) due to increased physical monitoring. However, other clinicians did not anticipate *‘much of an impact on us staff wise’* (P9), while yet others saw opportunities for staff to gain *‘another string to their bow’* (P6) through working with more severe presentations. One clinician worried about the effect of short admissions in the stepped‐care DP arm on IP staff.

Would we still get to see the bits of the job that make it a rewarding job for us? …what does that mean for staff morale and job retention and staffing in the future? (P15)

Some clinicians speculated whether trial duties, such as record‐keeping and data collection, might add *‘extra burden*’ (P20) to staff. Several clinicians interviewed towards the end of the trial noted that clearly defining staff roles in the trial helped circumvent this issue, with one clinician reporting ‘*as soon as we allocated, ‘You're going to be doing interviews, you're going to be doing allocation,’ it just flew’* (P24). In‐service DAISIES trial research champions were viewed positively due to their *‘links to the wider team’* (P4), with clinicians viewing them as *‘someone to go to with questions*’ (P12).

#### 3.2.3 The importance of communication between clinical and research teams

Many clinicians praised the clear channels of communication between the DAISIES research team and clinicians, which helped to *‘keep it on people's mind’* (P23) and *‘take the anxiety a little bit away for the staff’* (P5) at the beginning of the trial. Clinicians typically described the support they received as *‘fantastic’* (P3) and *‘accommodating’* (P24). However, communication was not always ideal, and a few clinicians reported feeling *‘out of the loop’* (P10). Some described the trial as *‘confusing’* (P19) and *‘very complicated’* (P4), noting difficulties understanding what was required of them to implement the trial.

Trial days and learning events, where clinicians from all sites met to discuss the trial, the challenges they were facing, and strategies to problem‐solve these, were viewed positively by most clinicians. The opportunities for information‐sharing and research team support were valued, as was the communication with other sites.

The days when everyone got together I think were really useful in enhancing motivation because it makes people feel part of something bigger (P26)

### 3.3 Theme 3: Concerns around the clinical management of participants

#### 3.3.1 Worries of appropriateness for level of acuity

Many clinicians worried about the appropriateness of trial treatment pathways for the severity of patient presentations. This worry primarily concerned inpatients, whose *‘needs are very complex and it's not just about their eating disorder’* (P8). Several clinicians also expressed concern that the trial may validate or invalidate patients' EDs.

If you then randomise somebody else and they don't get in‐patients, they might think that they're not ill enough or that they don't deserve treatment (P6)

For the IP arm, where the aim was for patients to remain on the ward until they reached a healthy BMI or got as close to that as possible, several clinicians noted the risk of institutionalisation, as the environment *‘can be very safe and containing and attachments get made with staff’* (P6). The suitability of IP treatment as a first‐line for younger patients with shorter illness duration was also questioned by some clinicians.

I'm thinking if I had an 18‐year‐old, first episode of illness… I would definitely want to keep her off the inpatient unit as a first choice (P10)

For the stepped‐care DP arm, clinicians commonly wondered about the level of engagement from patients who may otherwise have been offered IP care or were stepped down to DP quickly, as day treatment *‘requires the person to really want to engage and want to work with us’* (P14).

Despite these concerns, several clinicians suggested that the treatment arms would be beneficial for patient care in principle, particularly the stepped‐care DP arm. Stepping‐down patients to day services earlier than currently practiced was felt to *‘make that transition from the ward to the community smoother’* (P20) and to help with patient motivation in IP treatment, as they would have *‘a clear goal of being a day patient’* (P2) from the outset, and *‘feel more connected with their everyday lives and have more, kind of, autonomy and responsibility’* (P4). An earlier return to the home environment was consistently viewed positively, allowing for support from loved ones and maintenance of treatment gains.

Hopefully, consolidate those skills and get better overall treatment outcomes in terms of preventing future admissions and in terms of recovery (P20)

#### 3.3.2 Increasing risk, increasing anxiety

Clinicians often worried about increased risk and how best to manage this, particularly for patients in the stepped‐care DP arm.

Day services can’t hold the severity of, sometimes, the risk… that the inpatient unit can hold. (P17)

Day service clinicians interviewed early in the trial often reported feeling *‘loads of anxiety’* (P5), *‘a lot of fear’* (P6) and some *‘resistance to working with the more physically compromised patients’* (P11) coming to their services via the stepped‐care DP pathway. Carers of participants were also perceived to worry by some clinicians, as *‘the relief about having someone else taking care of the situation’* (P1) that IP care provides would be lost, and carers would have to take on ‘*responsibility they weren't expecting to have’* (P10). Several clinicians reported a dislike of overriding day services' typical admission criteria, reflecting a greater concern of research overriding clinical practice.

Pre the DAISIES way of thinking, in terms of treatment, [this] would not have been considered for that patient at that point in time … I get why it happens, but it does feel uncomfortable when you hear it. (P10)

Several clinicians reported not recruiting certain patients because they *‘felt the risk was too high… to even consider the potential option of stepping down early’* (P4). However, one DP clinician conveyed that their anxieties were never realised, and that ‘*it felt very much like we were just working with somebody that would come through our usual channel anyway’* (P4).

#### 3.3.3 Perceived impact on patient dynamics in services

Many clinicians commented on the potential impact that the DAISIES trial might have on patient dynamics within their services. Mixing illness severities was a common concern for both those less and more ill in DP and IP settings. IP clinicians additionally typically worried about the possibility of participants picking up *‘behaviours from other patients’* (P20), especially for patients new to IP services. Some DP clinicians felt that being around higher‐weight patients would be positive for the recovery of DAISIES participants.

People that are struggling more might be able to be pulled up a little bit more by the others, it might be, kind of, more motivational (P4)

However, others worried about the negative impact on current patients of being around lower‐weight patients.

You can see somebody else struggling a lot, it can impact on your own recovery. It's really hard (P19)

Clinicians often initially speculated that patients not in the trial may be ‘*envious’* (P8) of those that were, and that random allocation to differing treatment pathways *‘will create the perception of injustice’* (P3). Accordingly, clinicians expressed concerns over how best to manage this, with one clinician expressing that ‘*it would definitely add a more difficult dynamic for the staff’* (P7). Despite these concerns, one clinician interviewed after trial closure shared that ‘*I don't think it did affect the dynamics that much’* (P4).

### 3.4 Theme 4: Systemic capacity and capability issues

#### 3.4.1 National bed availability concerns

Clinicians commonly raised concerns over the availability of spaces in intensive treatment settings across the UK. This was a particular concern for IP services, which *‘rarely have empty beds’* (P15) and are pressured to *‘discharge patients as early as possible to make space for those with very low BMIs’* (P3). Consequently, many clinicians questioned how viable it was for a fluid stepped‐care model to be implemented, and whether beds would be available in IP services for those with higher BMIs.

Given the national shortage of beds, it will be very difficult, for example, a patient who's BMI 12, compared to a BMI 15.5, to justify offering the bed to the 15.5 and asking the 12 to wait for longer. (P8)

Aside from beds, clinicians raised several other resource scarcity concerns, most notably *‘staffing issues’* (P25) and financial pressures, that further stretched the capability of services to prioritise and implement DAISIES.

We are in the predicament to perform a study in an already under‐resourced process with the ultimate goal to save further money, and this is not possible to strike a balance under these circumstances where the pressure is already so much. (P3)

#### 3.4.2 Difficulty implementing the DAISIES trial in pathway logistics

Implementing the DAISIES trial into intensive treatment pathways was often described as challenging. Bed availability factored into these concerns, with clinicians wondering how best to accommodate participants given systemic pressures.

If we were keeping spaces free for DAISIES patients but then nobody was allocated, then how do you justify having some spaces and then a waiting list (P4)

One clinician felt that pressures might be so great that participants would have to be admitted to another service due to lack of capacity, worrying that *‘keeping a cohesive treatment plan might be quite difficult’* (P2). Continuity of care was also often noted as an issue for the stepped‐care DP pathway, as intensive services in the same NHS Trust were often said to be *‘quite separate teams’* (P18). The need for *‘better communication’* (P17) and *‘liaison work’* (P11) between intensive services were highlighted.

One of the hopes I think I’ve got is that this will help us set up a more streamlined pathway… rather than different groups of professionals re‐learning about them each time they move to a different bit of the service (P15)

Provider collaboratives, where several NHS Trusts accept patients through a single point of referral and pool their treatment facilities, were felt by several clinicians to further complicate implementation as eligible patients came from large geographical areas and some would struggle to travel to DP treatment if randomised to the stepped‐care pathway.

I've had patients admitted from all over the region, and so there's hardly anyone being admitted to my ward that actually can commute to our day treatment service (P22)

Many clinicians also believed that, in principle, the stepped‐care DP pathway should have been a good fit for service pathways as *‘it's already normal practice that we do stepped‐care’* (P8), although some felt ‘*quite a significant shift’* (P26) in boundaries was required to accommodate the step‐down of lower‐BMI patients.

### 3.5 Theme 5: Covid‐19 disrupting implementation

#### 3.5.1 Covid‐19 reducing the recruitment pool

Many clinicians discussed how recruitment from IP services, already noted to be challenging, was negatively impacted by the Covid‐19 pandemic and associated infection control measures. Service capacity reduced nationwide, while *‘demand grew’* (P4), with one clinician stating that *‘we've tripled our referrals'* (P25). Some clinicians noted that the mounting demand for IP services coupled with increasing pressure to discharge patients earlier to accommodate it *‘meant that the people coming to in‐patients… were really sick’* (P26), and so either not deemed appropriate for the trial due to risk or were difficult to engage. This was recognised in TMG meetings towards the end of the trial as one of the *‘distinct reasons why recruitment isn't working’* (TMG15).

Hospital admissions with reductions in beds were just so much more pressurised… we didn't have enough referrals suitable for DAISIES (P24)

The reduced capacity and growing waiting lists of services were also felt by many to negatively impact the already‐challenging implementation of the stepped‐care pathway. Overburdened services could not guarantee places, leading to a situation described by one clinician where ‘*people were allocated to the day‐care arm but stayed for 2 months or even longer in* [in]*patients’* (P3) before being stepped down.

#### 3.5.2 Covid‐19 changing the format of service provision

The most discussed impact of Covid‐19 on the implementation of the DAISIES trial was the changes brought to intensive ED services nationwide. This was especially relevant to DP services, which either offered reduced programmes, moved provision online, or closed.

We could only have people for short days, and in smaller numbers, and less acute. (P26)

Those offering virtual care often expressed the opinion that virtual provision was ‘*not comparable to the intensity of a normal day patient programme’* (P26), which was felt by one clinician to affect the stepped‐care DP pathway, as ‘*you don't want to take the risk of someone doing a virtual day treatment with a very low BMI’* (P22). For IP services, the standard of care was also seen to change due to infection control restrictions.

There’s been a lot of restrictions like people not being allowed to have visitors, not being allowed to have home leave. (P20)

Changes to service provision caused by Covid‐19 were typically seen as unequal across NHS Trusts. Several clinicians reflected that there was already ‘*so much difference between services and how different services are set up’* (P23), so this gap in provision only widened.

There is a heterogeneity in day service design, especially in a post‐pandemic world where many treatments have moved online. (TMG16)

Given these significant changes to provision, TMG minutes document amendments to trial protocol to ‘*accept a blended delivery of day patient treatment as part of the trial’* (TMG3) and to postpone the internal recruitment pilot phase of the trial, as many of the trial sites had closed their day services and therefore could not implement the treatment pathways and recruit.

Despite adaptations made to trial design, the difference in intensive service provision nationwide and the departure from traditional care that Covid‐19 necessitated (and the uncertainty over whether this would return), made many question whether DAISIES was still *‘going to be useful now’* (P26).

The unforeseen effects of the pandemic have created a situation in which the assumptions that were made during the initial study application have changed, and in all likelihood will not return (TMG16)

#### 3.5.3 DAISIES no longer a priority for clinicians

Clinicians interviewed after trial closure reflected on how the pressures brought by Covid‐19 on services led to changes in mindset amongst themselves and their staff regarding implementing the DAISIES trial. Clinicians commonly reported that other responsibilities *‘became much more colossal in the context’* (P26) and that the trial ‘*would have been probably the last thing on people's minds’* (P4), due to the severe impact on services described above, and the added stresses to staff's personal lives. The lack of physical researcher presence in services exacerbated this, as the DAISIES trial felt *‘more abstract with everything being online’* (P18).

When reflecting on the reasons behind closure, several clinicians conveyed a perception that Covid‐19 was the key reason for the ultimate failure of the trial.

Overall the trial couldn't recruit enough people in a meaningful way and the randomisation became much more blurred. Covid‐19 killed it. (P26)

### 3.6 Application of the NASSS framework

The application of the NASSS framework to the interpretive themes can be found in Table 4. Applying the NASSS suggests that all domains aside from the technology and the value proposition were characterised by barriers to implementation and classified as complex. Of these, the adopters, organisation, and wider system domains had the greatest number of barriers, indicating the greatest challenges to implementation. Several subthemes, such as ‘Increasing risk, increasing anxiety’ were present across multiple domains.

**TABLE 4:** Classification of themes by NASSS domain and as barriers or facilitators to implementation, and classification of overall domain complexity.

Domain	Complexity	Relevant themes	Description	Classification
1. The condition or illness	Complex	Difficulty pitching the trial to patients	Concerns over mental capacity; ambivalence and anxiety	Barrier
		Worries of appropriateness for level of acuity	Severity of patient presentations	Barrier
		Covid‐19 reducing the recruitment pool	Increased presentation severity for patients within services	Barrier
2. The technology	Simple	The importance of communication between clinical and research teams	Support required for implementation described to be strong	Facilitator
3. The value proposition	Complicated	The perceived appeal of DAISIES to participants	Altruistic motivation; dislike of assessments and randomisation	Facilitator/barrier
		Strong preference for day treatment	Seen as both positive and negative	Facilitator/barrier
		The appeal of changing standard practice	Addressing necessary questions; opening up discussion about standard practice	Facilitator
		Worries of appropriateness for level of acuity	Belief that the stepped‐care arm would be beneficial for patient care	Facilitator
		Increasing risk, increasing anxiety	Staff and carers concerned about patient safety; carer burden	Barrier
4. The adopter system	Complex	Difficulty pitching the trial to patients	Concerns around the acceptability of the trial to patients	Barrier
		Strong preference for day treatment	Preference as deterrent for participation	Barrier
		Changes in workloads and roles	Positive and negative appraisals of changing staff roles and workloads; defining research‐related roles aided implementation	Facilitator/barrier
		Worries of appropriateness for level of acuity	Concerns around institutionalisation and engagement of patients in treatment arms	Barrier
		Increasing risk, increasing anxiety	Worries around increased role of carers	Barrier
		Perceived impact on patient dynamics in services	Seen as both a positive and negative impact for patients; concerns over how to manage dynamics	Facilitator/barrier
		DAISIES no longer a priority for clinicians	Covid‐19 service pressures more important than implementing DAISIES	Barrier
5. The organisation	Complex	Increasing risk, increasing anxiety	Changes to the types of presentations worked with	Barrier
		National bed availability concerns	Resource scarcity concerns hindering implementation	Barrier
		Difficulty implementing the DAISIES trial in pathway logistics	Concerns around capacity of and communication between services	Barrier
		Covid‐19 changing the style of service provision	Significant changes to service operation and provision	Barrier
6. The wider context	Complex	National bed availability concerns	Nationwide capacity concerns prior to Covid‐19	Barrier
		Difficulty implementing the DAISIES trial in pathway logistics	Provider collaboratives complicating recruitment	Barrier
		Covid‐19 reducing the recruitment pool	Reducing capacity and increasing demand impacting stepped‐care implementation	Barrier
		Covid‐19 changing the format of service provision	Service closure and unequal provision across NHS Trusts	Barrier
7. Embedding and adoption over time	Complex	Covid‐19 disrupting implementation	The disruption brought by Covid‐19 decreased organisational capacity for implementation and innovation	Barrier

## 4 DISCUSSION

This qualitative study investigated stakeholders' views and experiences of implementing the DAISIES trial within intensive ED services. The themes and subthemes identified in the analysis span from the individual level (e.g. patient preference factors) to the systemic level (e.g. service capacity), and suggest that the greatest challenges in implementation existed with the adopters, organisational systems, and the wider socio‐political context.

The barriers identified in the adopter system domain chiefly concern patients and clinicians. Patient‐related barriers primarily surrounded the acceptability of the treatment arms, which was further complicated by aspects of ED symptomatology, such as high ambivalence. This is consistent with previous literature suggesting patient treatment preference as a key recruitment barrier in randomised controlled trials (RCT; Donovan et al., 2014; Elliott et al., 2017; McDermott et al., 2021). Additionally, AN populations are noted to be challenging to recruit and retain in RCTs due in part to high ambivalence and low treatment acceptability (Halmi, 2008; Halmi et al., 2005; Vinchenzo et al., 2022; Watson & Bulik, 2013). A potential solution may be to better accommodate patient preferences in the conduct of trials, either during the recruitment ‘pitch’ (Mills et al., 2011) or in research design (Loeb et al., 2020).

Clinician‐related barriers involved changes to staff modes of working and concerns over patient appropriateness for trial interventions. Both have been previously identified as common barriers to recruitment in trials (Briel et al., 2016; Bucci et al., 2015; Elliott et al., 2017; Howard et al., 2009; Rooshenas et al., 2016), and represent a larger tension between clinical and research roles for recruiting clinicians (Borschmann et al., 2014). This tension was commonly expressed around decision‐making for stepping‐down patients from IP to DP services. Previous authors have suggested that trial research processes should be well‐integrated into the existing working patterns of clinicians (Team et al., 2018), especially within IP services (Jacobsen et al., 2022), since clinical responsibilities will always take priority over those of research (Elliott et al., 2017). These previously‐disseminated challenges were however known to the DAISIES team, being comprised of a group of experienced trialists, and several mitigating strategies were implemented (İnce et al., 2023) including assigning research champion roles to clinicians, shown previously to improve recruitment within mental health service contexts (Oduola et al., 2017).

In parallel to the implementation barriers identified above, clinicians positively appraised the idea of the DAISIES trial. There were several facilitators in the value proposition domain, including staff belief in the importance of the trial, and a perception that altruism would motivate participation, consistent with literature on patient‐centred enablers of recruitment (Houghton et al., 2020). Previous research has identified positive opinions of a trial amongst recruiting staff, good communication, and supportive relationships between research and clinical teams as facilitators of trial success (Borschmann et al., 2014; Jacobsen et al., 2022; Peckham et al., 2018; Team et al., 2018). All were present in the DAISIES trial; however, the utility of these facilitators as well as the mitigation strategies applied by the DAISIES team appear to have been overshadowed by other implementation barriers.

Both the organisation and wider system domains were characterised by complexity and barriers to implementation. Barriers in the organisation domain primarily concerned low service capacity and difficulty implementing the stepped‐care DP pathway in service structures. Barriers in the wider system domain concerned the impact of Covid‐19 on the intensive ED healthcare system. Regarding service demand, hospital admissions for EDs were increasing prior to Covid‐19 without an appropriate rise in funding for adult ED intensive services (Ayton et al., 2022; NHS Digital, 2020). During the pandemic, specialist ED services experienced further increases in admissions, referrals, and symptom severity, concurrent with service closure and capacity reductions, both in the UK and internationally (Ayton et al., 2022; Hyam et al., 2022; Linardon et al., 2022; NHS Digital, 2020). In the context of the implementation of the DAISIES trial, this systemic pressure diminished the recruitment pool and services' capacities to implement timely stepped‐care. The introduction of provider collaboratives, partnerships between healthcare providers that aim to improve access to specialist services within their catchment areas (NHS England, 2021), additionally may have hindered implementation due to inequitable access to DP care post‐discharge. This reflects broader concerns of geographical inequality in ED care across provider collaboratives (Viljoen & Ayton, 2021). Finally, the impact of Covid‐19‐related infection control restrictions created a unique challenge for the DAISIES trial, facilitating an unpredicted pivot to virtual DP provision. Aside from some promising preliminary data (Obiekezie et al., 2022; Plumley et al., 2021), the effectiveness and efficacy of virtual DP provision are largely unknown and should be investigated in future research.

Organisational factors have ramifications for individual‐level areas of implementation. Primarily, systemic overburden contributes to increased clinical workloads and decreased available time for research, both of which have been previously identified as barriers to recruitment and research implementation in clinical services (Borschmann et al., 2014; Couturier et al., 2018; Fletcher et al., 2012) and as contributors to the tension between clinical and research roles (Elliott et al., 2017; Team et al., 2018). The negative impacts of under‐resourcing and overburden on ED patient safety and clinician experiences have been previously reported (Johns et al., 2019; Viljoen & Ayton, 2021; Webb et al., 2022a), but this study is the first time the negative impacts on research implementation in a UK healthcare context have been qualitatively explored. The results suggest that while organisational barriers to implementing the DAISIES trial existed prior to Covid‐19, the impact of the pandemic strengthened these barriers whilst creating unique challenges. More generally, the results indicate that systemic overburden and underfunding have limited the capacity for research and innovation in intensive ED services at a time when they are most needed.

### 4.1 Strengths and limitations

This study is a novel application of the NASSS framework to a clinical trial, conceptualising the trial as the technology to be implemented. The theory provided a useful organising framework for understanding implementation. The study used a wide variety of data from across the DAISIES trial, and efforts were made throughout to ensure the reliability and transferability of results, such as engaging in researcher reflexivity, multiple researcher coding, and the use of the NASSS framework. However, the data are limited in that they omit participant and carer accounts, and the results of the analysis are bound to the context of intensive ED services in the UK, limiting transferability. However, the impact of Covid‐19 on ED services has been felt similarly internationally (Linardon et al., 2022; Weissman & Hay, 2022), and many of the reported barriers to implementation are echoed by other qualitative research in different countries and healthcare contexts. Another limitation is that in the first period of interviews, clinicians were told that their answers may potentially benefit study implementation (see Appendix 2), which may have biased responses.

### 4.2 Conclusion

This study provides in‐depth insight into the challenges faced in implementing the DAISIES trial within UK‐based intensive ED services. The findings echo and expand upon previous qualitative research into trial recruitment difficulties, placing particular emphasis on the top‐down impact of systemic‐level implementation challenges on individuals. The findings imply that future trials in intensive ED services need to accommodate patient preference to improve acceptability, foster supportive relationships between clinicians and researchers to help prioritise research, and optimise time spent on research within clinical teams through aligning research processes with practice and clearly designating responsibilities amongst clinical staff.

The specific systemic challenges encountered in the DAISIES trial demonstrate the profound impacts of an underfunded and overburdened system on research implementation, underscoring the need for investment into adult ED services in the UK as has previously been suggested regarding improving care (Viljoen et al., 2022). The results suggest that currently, conducting an RCT of the scope and magnitude of the DAISIES trial may not be possible within intensive adult ED services in the UK, and alternative research designs should be explored, such as naturalistic longitudinal studies. Though the DAISIES trial failed, research into less‐restrictive treatment alternatives for adult EDs remains necessary.

## CONFLICTS OF INTEREST STATEMENT

The authors have no conflicts of interest to declare.

## Supporting information

Supplementary Material

Supplementary Material

Supplementary Material

## Data Availability

The data corpus of this study is not publicly available but can be supplied by the corresponding author following reasonable request.
