# Supplementary material for: Autopsy of a failed trial part 1: A qualitative investigation of clinician's views on and experiences of the implementation of the DAISIES trial in UK‐based intensive eating disorder services
Source: Eur Eat Disord Rev. 2023 Mar 23;31(4):489–504. doi: 10.1002/erv.2975 (PMC10946575; doi:10.1002/erv.2975)
Supplement: Supplementary file 1 — Supplementary Material [file ERV-31-489-s001.docx]

**Appendix 1**

*Table 1: Dates of semi-structured interviews and participant ID of interviewee*

| **Interview Date** | **Participant** |
| --- | --- |
| 22/05/2020 | P1 |
| 05/06/2020 | P2 |
| 29/06/2020 | P3 |
| 03/07/2020 | P4 |
| 10/07/2020 | P5 |
| 13/07/2020 | P6 |
| 27/07/2020 | P7 |
| 13/10/2020 | P8 |
| 09/11/2020 | P9 |
| 12/11/2020 | P10 |
| 26/11/2020 | P11 |
| 09/12/2020 | P12 |
| 02/02/2021 | P13 |
| 12/02/2021 | P14 |
| 16/02/2021 | P15 |
| 26/02/2021 | P16 |
| 06/04/2021 | P17 |
| 06/04/2021 | P18 |
| 09/04/2021 | P19 |
| 01/06/2021 | P20 |
| 04/05/2022 | P4 |
| 14/06/2022 | P26 |

*Table 2: Dates of focus groups and IDs of participants*

| **Focus Group Date** | **Participants** |
| --- | --- |
| 21/04/2022 | P3; P8; P21; P22 |
| 03/05/2022 | P23; P24; P25 |

*Table 3: Dates of Trial Management Group and Trial Steering Committee meetings and related in-text identifier*

| **TMG/TSC Date** | **Identifier** |
| --- | --- |
| 12/03/2020 | TMG1 |
| 14/05/2020 | TMG2 |
| 09/07/2020 | TMG3 |
| 13/08/2020 | TMG4 |
| 08/10/2020 | TMG5 |
| 12/11/2020 | TMG6 |
| 10/12/2020 | TMG7 |
| 14/01/2021 | TMG8 |
| 11/02/2021 | TMG9 |
| 11/03/2021 | TMG10 |
| 08/04/2021 | TMG11 |
| 13/05/2021 | TMG12 |
| 08/07/2021 | TMG13 |
| 09/09/2021 | TMG14 |
| 11/11/2021 | TMG15 |
| 06/12/2021 | TSC1 |
| 11/01/2022 | TMG16 |
| 19/03/2022 | TMG17 |
| 30/03/2022 | TMG18 |

*TMG = Trial Management Group, TSC = Trial Steering Committee*
