# Supplementary material for: Autopsy of a failed trial part 1: A qualitative investigation of clinician's views on and experiences of the implementation of the DAISIES trial in UK‐based intensive eating disorder services
Source: Eur Eat Disord Rev. 2023 Mar 23;31(4):489–504. doi: 10.1002/erv.2975 (PMC10946575; doi:10.1002/erv.2975)
Supplement: Supplementary file 3 — Supplementary Material [file ERV-31-489-s003.docx]

**Appendix 3: Topic guide for clinician interviews and focus groups conducted between April – June 2022**

We’d like to begin by asking you about your feelings surrounding the trials closure. We will then move onto a discussion of your experiences of different aspects of the study such as recruitment, implementation etc.

So, first of all, hearing that the trial is to be closed down, what thoughts and feelings went through your head?

- *Was it a surprise? Was it expected? A relief? Entirely understandable if it was all of these and more*

Casting your mind back to when you first became involved with DAISIES: What were your first impressions of the DAISIES trial? **(Adopter system)**

- - *In your opinion, what were the benefits of the DAISIES trial?*
  - *How have your opinions about DAISIES changed over time?*
- How did other professionals in your service feel about DAISIES?
  - *Did this change over time?*

What was your experience of implementing/running the DAISIES trial in your service(s)? (**Healthcare organisation)**

- Did you have to change anything in your service in order to accommodate the requirements of the trial?
- Thinking about implementing/running the trial: What went well, what was more challenging?
  - *How did these change over time?*
- Were there any NHS structures (e.g. provider collaboratives), people (e.g. senior management) or other organisational factors that helped or hindered implementation of the trial in your service?

What was your experience of implementing the stepped-care pathway of the trial like? **(Technology)**

- How did implementing the stepped-care pathway affect your service provision?
- How did the stepped-care approach fit in with your existing infrastructure?
  - *How did you feel about the risk-assessment tool?*
- Were there any unexpected challenges in implementing the stepped-care approach?
  - *Did these change over time, and did new challenges emerge?*
- How do you think patients perceived the stepped-care pathway and do you think this changed over time?

What was your experience of the in-patient treatment pathway in the trial, i.e. where the emphasis was on keeping patients as inpatients for as long as needed to maximise weight gain/recovery?

- Did this mean any changes to your usual practice?
  - *Did this change over time, and did new challenges emerge?*
- How do you think patients perceived the inpatient pathway and do you think this changed over time?

What were your experiences of approaching and recruiting patients for the trial like? **(Adopter system/value proposition/condition)**

- What was your opinion of conducting a clinical trial with this patient population?
- What do you think the main challenges for recruitment were?
- How do you think patients felt about participating in the trial when they were approached?
  - *What positives/negatives do you think they may have seen about participation?*
- Do you think the DAISIES trial impacted patient dynamics within your service(s) in any way, if so how?

How did Covid-19 impact supporting/implementing the trial in your service(s)?

- How do you think Covid-19 impacted the trial as a whole?
  - *In terms of recruitment, implementation within the service*

Overall, could anything have been done differently or better, from either our end or yours?

What are the main learning points for us at this stage?

How can we best approach future research in this area?

Finally, is there anything else you’d like to add that we haven’t covered?
